# The influence of night shift work and associated factors on serum uric acid in aircraft maintenance workers

**DOI:** 10.1186/s12889-024-18849-4

**Published:** 2024-07-23

**Authors:** Huahuang Dong, Yanlin Cao, Xiaowen Ding, Tenglong Yan, Chu Zhou, Mingli Bi, Huining Wang, Xue Wang, Jue Li

**Affiliations:** 1 Beijing Institute of Occupational Disease Prevention and Treatment, Beijing, China; 2 School of Population Medicine and Public Health, Chinese Academy of Medical Sciences and Peking Union Medical College, Beijing, China; 3 Shanxi Medical University, Taiyuan, China

**Keywords:** Shift work, Serum uric acid, Influencing factors

## Abstract

**Background and objective:**

The prevalence of 12-hour shift work is increasing in various occupations. Shift work has been linked to circadian rhythm disruption, which may lead to hormonal changes and metabolic disorders, including alterations in glucose, lipid, and purine metabolism. Despite this, there is limited research on the potential connection between work shifts and abnormal serum uric acid (SUA) levels. Furthermore, the factors that contribute to abnormal SUA levels in shift workers are not well-understood. Therefore, this study aimed to analyze the SUA levels of shift workers employed in an aircraft maintenance company, investigate the potential association between shift work and SUA levels, and explore the factors that may influence abnormal SUA levels in shift workers.

**Methods:**

A total of 2263 male workers from an aircraft maintenance company were included in this study using the cluster sampling method. The workers were divided into two groups based on their working shifts: night shift (*N* = 1047, 46.27%) and day working (*N* = 1216, 53.73%). A survey was conducted between April 1st and June 30th, 2022 to gather information on work, lifestyle, physical examination results, and other relevant factors. The survey included a self-designed demographic information questionnaire to collect data on workers’ characteristics, medical history, years of employment, smoking and drinking habits, and main lifestyle behaviors. The workers’ SUA levels were measured using uricase colorimetry. One-way ANOVA was used to compare the difference in the abnormal detection rate of SUA between the two groups, and multi-factor logistic regression analysis was used to identify the factors that influence abnormal SUA levels.

**Results:**

The study indicated that 48.9% of night shift workers and 43.8% in the regular day workers had abnormal SUA levels, with a significant difference between the two groups (*χ*^2^ = 6.125, *P* = 0.013). Factors such as circadian rhythm type, shift work, age, the taste of diet, type of diet, smoking, overweight or obesity based on body mass index (BMI), concentration of urine creatinine (CREA), total cholesterol, triglyceride, and low-density lipoprotein cholesterol were found to be correlated with SUA abnormalities (*P* < 0.05). The risk of developing SUA abnormalities was found to be higher in individuals with an intermittent (*OR* = 1.34, 95% CI: 0.83–2.12, *P* < 0.05) or evening circadian rhythm type (*OR* = 1.45, 95% *CI*: 0.86–2.43, *P* > 0.05) compared to those with a morning type. Additionally, factors such as night shift work, a high-sodium diet, smoking, a diet high in meat and low in vegetables, being overweight or obese, and higher levels of CREA were also found to increase the risk of developing SUA abnormalities. The study also revealed a significant dose-response relationship between BMI and abnormal uric acid levels. After controlling for other factors, the risk of developing SUA abnormalities was found to be 1.18 times higher in the night shift work group than in the day work group (*OR* = 1.18, 95% *CI*:1.02–1.34, *P* = 0.01).

**Conclusion:**

Shift work has been linked to a higher risk of developing SUA abnormalities, and there are several factors that may contribute to this risk. To prevent diseases, it is recommended that enterprises implement better health monitoring and management practices for shift workers.

## Introduction

An increasing number of enterprises and service industries, such as mining, metallurgy, manufacturing, transportation, and healthcare services, have implemented shift work schedules for their employees [1, 2]. According to statistics, shift workers now make up approximately 20% of the global labor force. However, Studies have shown that this deviation from traditional daily routines disrupts circadian rhythms and can lead to sleep disorders, resulting in a range of physiological and psychological issues that significantly impact individuals’ overall health [3–5].

Research has also indicated that shift work has a significant effect on various biomarkers [6], and is associated with metabolic diseases [7], cardiovascular diseases [8], mental illnesses [9], cancer [10], and more. One important biomarker affected by shift work is SUA, the end product of purine metabolism in the body. A previous review focused on several diseases and found strong links between SUA and oxidative stress and circadian rhythm processes, including aging, cardiovascular diseases, cancer, metabolic syndrome, and neurodegenerative disorders [11]. Normally, SUA levels are maintained in a dynamic balance of excretion and production in the human body. However, when this balance is disrupted, it can lead to increased blood levels of uric acid, which can raise the risk of renal damage and coronary atherosclerosis [12, 13]. Unfortunately, uric acid abnormalities often go unnoticed in the early stages, as they typically appear without symptoms. This can result in delayed or inadequate treatment for patients with hyperuricemia, leading to further complications [14]. While some studies have explored the correlation between shift work and SUA levels [15], there is a lack of large population studies with diverse sources of the population. Therefore, the main objective of this study is to investigate the correlation between abnormal SUA levels in aircraft maintenance personnel and shift work, identify potential influencing factors, and provide a scientific basis for promoting the occupational health of this population.

## Study design

### Study population

We conducted a questionnaire survey among 3000 aircraft maintenance personnel to examine the lifestyle of participants in Beijing, China. After applying inclusion and exclusion criteria, we collected a total of 2602 questionnaires, resulting in high recovery rate of 86.7%. The inclusion criteria required participants to be between the ages of 18 and 60, free of major illnesses in the past 6 months, and have obtained clearance from the Ethics Committee of the Beijing City Institute of Chemical Industry Occupational Disease Prevention and Treatment. Additionally, participants were required to have a clear understanding of the study and provide voluntary participation. The exclusion criteria included non-standardized questionnaires, incomplete survey items, and missing uric acid results.

All participants in this study work in two patterns: regular day working, from 8:00 to 17:00 on weekday and a rotational night shift working of “day shift-night shift-rest-rest”. Specifically, night shift working pattern refers to: the first day of work is from 8:00 to 20:00, and the second day is from 20:00 to 8:00 of the third day, and then a rest period from 8:00 of the third day to 8:00 of the fifth day. The working pattern cycle starts on the fifth day from 8:00. In this study, individuals who have been working in this shift system for over six months are considered night shift workers.

Two well-trained investigators collected the information and underwent independent data cleaning and analysis to ensure the quality of the data. The stability and consistency of the questionnaire were assessed by administering it to the same group of individuals at various intervals and comparing the results. The reliability and validity of the data were confirmed through expert review and pilot testing.

### Data collection

The questionnaire survey aimed to gather sociodemographic characteristics, occupational information, and lifestyle habits of the participants. This included age, date of birth, gender, and marital status, years of employment, shift work, exercise frequency, dietary habits, dietary patterns during night shifts, and circadian rhythm score.

Participants’ body height, body mass, and BMI were measured using standard methods. Blood pressure was also recorded. Biochemical indicators were assessed through venous blood and urine samples collected in the morning on an empty stomach. Uric acid levels were determined using the uricase colorimetry method, while total cholesterol (TC), triglyceride (TG), and CREA levels were assessed using the enzymatic method. Additionally, high-density lipoprotein cholesterol (HDL-c) and low-density lipoprotein cholesterol (LDL-c) levels were measured using the homogeneous method.

### Circadian rhythm scale

A scale was created to measure individuals’ preference for morning, intermittent, or evening circadian rhythms. The validity of the questionnaire has been shown 0.63 [16] in previous study. Its reliability and validity were good [17]. Cronbach’s α for this scale in our study was 0.637 and the reliability coefficients were in the range of 0.6-1, indicating that the scale had internal consistency. It assessed participants’ sleep and activity preferences. A higher score on the seven items indicates a stronger inclination towards being a morning person [18], less impact of circadian rhythm on mental state, and a lower overall score. Those with a total score of 14 or less were classified as evening types, those with a score between 15 and 21 were classified as intermittent types, and those with a score of 22 or higher were classified as morning types.

### Judgment criteria

According to the assessment criteria outlined in the fourth edition of the 2014 National Clinical Laboratory Operating Procedures, in conjunction with the reference range values established by our laboratory, SUA levels exceeding 420µmol/L were considered abnormal in adult males. Similarly, serum CREA levels exceeding 111umol/L, TC levels exceeding 5.20mmol/L, TG levels above 1.71mmol/L, and LDL-c levels above 111 mmol/L are also classified as anomalous. To better illustrate the BMI status of Chinese individuals, we used a lower BMI threshold than the WHO international standard. According to the established criteria [19], individuals with a BMI below 24.0 kg/m^2^ are categorized as having normal weight, those with a BMI ranging from 24.0 to 28.0 kg/m^2^ are considered overweight, and individuals with a BMI greater than or equal to 28.0 kg/m^2^ are classified as obese. The number of years of employment in their current position was divided into three groups: ≤9 years, 10–19 years, and ≥ 20 years.

### Statistical analysis

Statistical analysis was conducted using RStudio (version 2023.03.0 + 386; RStudio Team, 2023) and SPSS (version 27.0, SPSS Inc, Chicago, IL, USA) to compare variables between daily living habits and night shift work. Mean ± standard deviation (x̄± SD) was used and group comparisons were made using t-tests. In cases where the data did not follow a normal distribution, median (*P25*, *P75*) values were used and rank sum tests were performed. To explore the factors influencing abnormal uric acid levels, rates were used to represent variables such as night shift work, employment years, and night eating. The rates of categorical variables were described, and intergroup comparisons were made using the Chi-square test. Similarly, to investigate the risk factors associated with abnormal blood uric acid levels, rates were used to represent variables such as night shift work and night eating. The rates of categorical variables were described and differences between groups were analyzed using the Chi-square test. Binary multivariate logistic regression models were used to explore the risk factors associated with uric acid abnormalities. All statistical tests were two-sided and a significance level of *p* < 0.05 was used.

## Results

### Sociodemographic characteristics of the participants

The study included a total of 2,263 participants aged 19 to 60 years, with an average age of 34.1 ± 0.2 years. The participants were divided into two groups based on their work schedule: 1,047 were night shift workers (46.3%), and 1,216 were regular day workers (53.7%). Significant differences were found between the two groups in terms of age, length of service, smoking status, dietary preferences, and exercise habits (*P* < 0.05). Specifically, the median age of night shift workers and regular day workers was 32 and 34 years, and the median length of service was 9 and 12 years. 499 (47.7%) of night shift workers were smokers, while 487 (40.0%) of regular day workers were smokers. Similarly, 484 (46.3%) of night shift workers had a preference for salty diets, compared to 491 (40.4%) of day workers. In terms of exercise habits, 786 (75.1%) of night shift workers engaged in sports activities, while 951 (78.3%) of day workers did so. Additionally, 557 (53.2%) of the participants consumed tea or coffee, with 626 (51.5%) of day workers. Lastly, 92 (8.8%) of night shift workers used sleep aids, compared to only 44 (3.6%) in day workers (Table 1).

**Table 1:** Comparison of general characteristics of subjects

Variable		Night Shift workers(*n* = 1047)		Day workers (*n* = 1216)		*p*-value
		*N*	%	*N*	%	
Smoking	Yes	499	47.7	487	40.0	<0.001
	No	548	52.3	729	60.0	
Drinking	Yes	412	39.4	433	35.6	0.067
	No	635	60.6	783	64.4	
Dietary tastes	Light	369	35.2	484	39.8	0.017
	Salty	484	46.3	491	40.4	
	Partial to others	194	18.5	241	19.8	
Meat and vegetables combination	Reasonable mix	611	58.4	729	60.0	0.446
	Less meat and more vegetables	135	12.9	166	13.7	
	Less vegetables and more meat	301	28.7	321	26.4	
physical exercise	never	261	24.9	265	21.8	< 0.001
	1–3 times per quarter	227	21.7	203	16.7	
	2–3 times a month	153	14.6	172	14.1	
	1–2 times a week	280	26.7	327	26.9	
	3 times per week and more	126	12.0	231	19.0	
Tea/Coffee	Yes	557	53.2	626	51.5	0.414
	No	490	46.8	590	48.5	
Use sleep aids	Yes	92	8.8	44	3.6	<0.001
	No	955	91.2	1172	96.4	
Uric Acid (M ± SD)		422.65 ± 89.1		413.48 ± 93.58		0.018

a. Continuous variables that do not follow a normal distribution were tested using Wilcoxon rank sum test. The test statistic used is *Z*

b. For counting data, the Chi-square test is adopted, with the test statistic being the *χ*^*2*^ value

### Univariate analysis of factors influencing abnormal rate of uric acid in the aircraft maintenance population

The study found that the detection rates of abnormal uric acid levels was 48.9% among night shift workers and 43.8% among regular day workers. This difference was statistically significant (*χ*^2^ = 6.125, *P* = 0.013). The researchers also discovered that the rate of uric acid abnormalities varied significantly based on factors such as age, years of working, frequency of night shift eating, meat and vegetable consumption, BMI, levels of LDL-c, TG, TC, and CREA (all *P* < 0.05; see Table 2).

**Table 2:** Univariate analysis of abnormal uric acid [N (%)]

Variable	Normal uric acid		Uric acid abnormal		Total		*p*-value
	*N*	%	*N*	%	*N*	%	
Shift							0.013
Yes	535	51.1	512	48.9	1047	46.3	
No	684	56.3	532	43.8	1216	53.7	
Age							<0.001
≤ 29	340	47.6	375	52.4	715	31.6	
30–39	556	51.7	520	48.3	1076	47.5	
40–49	148	62.7	88	37.3	236	10.4	
≥ 50	175	74.2	61	25.8	236	10.4	
Employment years							<0.001
≤ 9	511	50.2	507	49.8	913	40.3	
10∼19	480	52.6	433	47.4	332	14.7	
≥ 20	228	68.7	104	31.3	1018	45	
Number of night shift meals							0.035
0	254	55.6	203	44.4	457	43.6	
1∼2	267	47.8	291	52.2	558	53.3	
≥ 3	14	43.8	18	56.3	32	3.1	
Combination of vegetarian							<0.001
reasonable	738	55.1	602	44.9	1304	58.55	
More vegetable	187	62.1	114	37.9	301	13.52	
More meat	294	47.3	328	52.7	622	27.93	
BMI							<0.001
Normal	418	69.4	184	30.56	602	26.7	
Overweight	489	55.4	393	44.6	882	39.1	
Obesity	309	40.1	461	59.9	770	34.2	
LDL-c							<0.001
Normal	1090	54.8	898	45.2	1988	89.6	
High	91	39.6	139	60.4	230	10.4	
TG							<0.001
Normal	844	59.8	567	40.2	1411	63.6	
High	337	41.8	470	58.2	807	36.4	
CHOL							<0.001
Normal	871	57.5	645	42.5	1516	68.3	
High	310	44.2	392	55.8	702	31.7	
CREA							<0.001
Normal	1209	54	1031	46.03	702	99.2	
High	7	38.9	11	61.11	18	0.8	
Night shift sleep duration							0.16
< 1 h	303	52.6	273	47.4	576	55	
1–3 h	170	47.2	190	52.8	360	34.4	
≥ 3 h	62	55.9	49	44.1	111	10.6	

To evaluate the normality of uric acid, one-way ANOVA was used. The findings showed that among 1219 patients with normal uric acid levels, the average score on the circadian rhythm scale was 18.15 ± 2.51, while in 1044 patients with abnormal uric acid levels, the average score was 17.93 ± 2.43. This suggests a significant difference between the two groups (*t* = 2.08, *P* < 0.05).

### Distribution of abnormal uric acid in night shift workers

The subjects were divided into four age groups (≤ 29 years, 30–39 years, 40–49 years, and ≥ 50 years) and three categories based on their years of working experience (≤ 9 years, 10–19 years, and ≥ 20 years). Within each age group, those who worked night shifts had a higher incidence of uric acid abnormalities compared to those who worked regular day shifts. This difference was statistically significant (*P* < 0.05). Additionally, individuals with less than 10 years of experience in the shift system showed a significantly higher rate of uric acid abnormalities compared to those on regular day shifts (*P* < 0.05, see Table 3).

**Table 3:** Distribution of abnormal uric acid in night shift workers

Variable		Night Shift Workers		Day Workers		*P*
		*N*	%	*N*	%	
Age/year	≤ 29	218	53.7	157	50.8	<0.001
	30–39	238	49.2	282	47.6	0.047
	40–49	33	41.3	55	35.3	0.005
	≥ 50	23	29.9	38	23.9	<0.001
Position Years/year	≤ 9	300	54.0	207	44.8	0.004
	10–19	185	45.3	248	49.1	0.286
	≥ 20	27	32.5	77	30.9	0.785
Total		512	50.53	532	43.8	

### Multivariate logistic regression analysis of related factors of uric acid abnormality

Multivariate logistic regression analysis was performed with the rate of SUA abnormalities as the dependent variable (abnormal = 1, normal = 0), and the total scores of circadian rhythm and individual factors listed in the table as independent variables.

The study revealed that various factors can influence uric acid abnormalities, including circadian rhythm type, night shift work, age, the taste of diet, type of diet, smoking, overweight or obesity based on BMI classification, CREA, TC, TG, and LDL-c (*P* < 0.05). The odds of developing SUA abnormalities were higher in individuals with an intermittent type (*OR* = 1.34, 95% *CI*: 0.83–2.12, *P* < 0.05) or evening type (*OR* = 1.45, 95% *CI*: 0.86–2.43, *P* > 0.05) of circadian rhythm than in those with a morning type. Other factors associated with an increased risk of developing SUA abnormalities included engagement in night shift work, high salt intake, smoking, preference for high-meat diets, being overweight or obese, and elevated levels of serum creatinine.

Furthermore, a significant correlation was found between BMI and uric acid abnormalities, with a clear dose-response relationship. After controlling for other variables, night shift workers were 1.18 times more likely to have SUA abnormalities than regular day workers (*OR* = 1.18, 95% *CI*: 1.02–1.34, *P* = 0.01). To further explore the association between abnormal uric acid levels and these factors, a binary logistic regression analysis was conducted (Table 4).

**Table 4:** Association between abnormal uric acid levels and impact factors

Variable	Abnormal uric acid						
	β	SE	Wald χ^2^	*P*-value	OR value	95%CI	
						Lower limit	Upper limit
Intermittent type	0.28	0.24	1.42	0.03	1.34	0.83	2.12
Evening type	0.37	0.26	1.96	0.16	1.45	0.86	2.43
Night shift	-0.21	0.09	6.12	0.01	1.18	1.02	1.34
age ≥ 50	-1.01	0.50	4.01	0.04	0.37	0.14	0.98
Salty diet habit	0.45	0.22	4.21	0.04	1.57	1.02	2.42
Diet with meat and low in vegetables	0.13	0.11	1.37	0.05	1.13	0.92	1.34
Smoking	0.237	0.11	6.42	0.01	1.27	1.06	1.526
BMI overweight	0.78	0.20	15.43	<0.001	2.19	1.48	3.24
BMI Obesity	1.39	0.21	44.28	<0.001	3.99	2.66	6.01
CREA ≥ 111umol/L	1.34	0.48	7.86	0.005	3.82	1.50	9.75
CHOL ≥ 5.2mmol/L	0.41	0.11	13.35	<0.001	1.51	1.21	1.88
TG ≥ 1.71mmol/L	0.72	0.098	53.8	<0.001	2.05	1.69	2.48
LDL-c ≥ 3.5mmol/L	0.72	0.15	24.5	<0.001	2.05	1.54	2.72

## Discussion

According to the International Labor Organization, night work is defined as working between 0:00 and 5:00. The night shift workers in this study followed a “day shift-night shift-rest-rest” pattern. Previous research has shown that more than 50% of night shift workers, especially those who work at night, experience insomnia and sleep difficulties. These issues are primarily caused by disruptions in biorhythms, which can lead to fatigue, psychological stress, social issues, and systemic illnesses [15]. In a recent review, the impact of external stressors on oxidative stress and damage in humans was summarized [20]. The review found that uric acid, a compound in the body, follows a significant circadian rhythm in healthy adults, with levels peaking early in the evening [6, 21]. This rhythm was also observed in other studies, with uric acid reaching its highest levels between 12:00 and 18:00 and its lowest levels between 00:00 and 06:00 [22, 23]. However, in another study, a different rhythm was reported [24].

The purpose of this study was to investigate the levels of SUA in night shift workers employed at an aircraft maintenance company and to identify potential factors that may contribute to the increased occurrence of SUA abnormalities.

The results of the study showed a higher rate of SUA abnormalities in the aircraft maintenance company compared to the general population in China (13.46-27.51%) [25–27], and in other countries [28–32]. There are several factors that could explain the elevated levels of SUA abnormalities. Firstly, the rate of abnormal SUA is higher among males than females [33], and the study participants were exclusively male employees. Secondly, aircraft maintenance workers were exposed to various complex factors in their work environment, including night shift work and other occupational hazards such as noise, high-altitude operations, dust, and kerosene, which could directly or indirectly impact SUA levels [34]. Finally, the company’s cafeteria offers a variety of high-fat and high-salt meals for employees, which may also contribute to the increased incidence of abnormal SUA levels [35].

In this study, the rate of SUA in night shift workers was significantly higher than that in regular day workers (*P* < 0.05). This finding was consistent with previous research [36], which showed a positive correlation between sleep duration and the likelihood of abnormal SUA. Specifically, a shorter sleep duration of sleep was found to be associated with a higher rate of abnormal uric acid. It is important to note that night shift workers face greater challenges in obtaining adequate and high-quality sleep than regular day workers [1, 37–39]. Correspondingly, the proportion of sleep aids used was significantly higher among night shift workers (*P* < 0.05). Furthermore, a lower circadian rhythm score indicates a reduced adaptation to the demands of night work [18]. The results of multi factorial analysis support the hypothesis that circadian rhythms play a significant role in the occurrence of abnormal uric acid. However, several studies have shown that individuals with higher circadian rhythm scores exhibit enhanced activity levels, suggesting that they are less affected by circadian rhythms in terms of their physical energy [40]. Further investigation is warranted to explore the tolerance to sleep-related night shift work.

In healthy individuals, approximately two-thirds of uric acid is excreted through the kidneys [41]. In our study, we found that higher levels of CREA increased the risk of developing SUA abnormalities. Several other studies have also demonstrated that the risk of developing SUA abnormalities was higher in individuals with abnormal level of CREA compared to those with normal level of CREA [42]. On one hand, elevated levels of SUA can lead to the formation of urate crystals, which may accumulate in the kidneys, triggering inflammation, endothelial dysfunction, and damage to the renal tubules. This can also lead to blockages in the renal tubules, further contributing to renal damage. On the other hand, impaired renal processing of uric acid plays a significant role in hyperuricemia development, including decreased glomerular filtration rate, increased reabsorption, and insufficient secretion of uric acid from the renal tubules [43, 44].

### Strengths and limitations of this study

This study has several notable strengths. Firstly, it is the first investigation to explore the potential association between night shift work and SUA abnormalities among aircraft maintenance workers. This is a significant contribution to the existing literature on the topic. Secondly, the study had a larger sample size than previous research, thereby reinforcing the credibility of our findings. Thirdly, we utilized a novel approach by evaluating the risk of developing SUA abnormalities in relation to the circadian rhythm score, which has not been reported in other studies and adds a unique perspective to our findings.

However, it is important to acknowledge the limitations of this study. Firstly, the participants were limited to aircraft maintenance workers, which may limit the generalizability of our findings to a wider population or other workplaces. Despite this limitation, it is worth noting that this group exhibited diverse characteristics that are the representative of the general population, including a high prevalence of overweight and obesity (39.1% and 34.2%, respectively). These rates are comparable to those reported in the same district for the years 2016–2017 [45]. Secondly, our study did not take into account important confounding factors, such as exposure to physical and chemical occupational hazards (noise, high-altitude operations, and aviation kerosene). This may have affected the results of our study. Thirdly, because our study was cross-sectional, we cannot establish a causal relationship between the prevalence of abnormalities in SUA and night shift work. However, it is reasonable to infer that night shift work may be an independent risk factor for developing SUA abnormalities.

Fortunately, we have access to comprehensive data for this population, which presents opportunities for further investigation. For example, future research could gather information on occupational hazard exposure, circadian rhythm of SUA levels [46, 47], mental well-being [48, 49], and longitudinal data. By analyzing the mediating role of these factors in the relationship between SUA levels and night shift work, we can better understand the detrimental effects of night shift work on the health of employees and potentially reduce it, enhancing their adaptability to night shift work.

## Data Availability

The datasets used and/or analyzed during the current study are available from the corresponding author upon reasonable request.

## Declarations

### Declaration

All participants confirmed their consent for their personal information and/or photographs to be published in an open-access journal.

## References

[1] Ganesan S, Magee M, Stone JE, et al. The impact of Shift Work on Sleep, alertness and performance in Healthcare Workers. Sci Rep. 2019;9(1):4635. 30874565 10.1038/s41598-019-40914-x PMC6420632

[2] Kwon P, Lundin J, Li W, et al. Night shift work and lung cancer risk among female textile workers in Shanghai. China J Occup Environ Hyg. 2015;12(5):334–41. 25616851 10.1080/15459624.2014.993472 PMC4400196

[3] PAPANTONIOU K, ESPINOSA A, BURGOS J et al. Night shift work, chronotype and prostate cancer risk in the MCC -Spain case -control study[J]. Int J Cancer 2014,137(5):1147–57. 10.1002/ijc.29400 25530021

[4] HUBLIN C, PARTINEN M, KOSKENVUO K, et al. Shift-work and cardiovascular disease: a population -based 22 -year follow -up study[J]. Eur J Epidemiol. 2010;25(5):315–23. 20229313 10.1007/s10654-010-9439-3

[5] VIMALANANDA VG, PALMER JR. Night -shift work and incident diabetes among African -american women[J]. Dia- betologia. 2015;58(4):699–706. 10.1007/s00125-014-3480-9 PMC4461435 25586362

[6] Kanabrocki EL, Murray D, Hermida RC, et al. Circadian variation in oxidative stress markers in healthy and type II diabetic men. Chronobiol Int. 2002;19(2):423–39. 12025934 10.1081/cbi-120002914

[7] Sooriyaarachchi P, Jayawardena R, Pavey T, et al. Shift work and the risk for metabolic syndrome among healthcare workers: a systematic review and meta-analysis. Obes Rev. 2022;23(10):e13489. 35734805 10.1111/obr.13489 PMC9539605

[8] Torquati L, Mielke GI, Brown WJ, et al. Shift work and the risk of cardiovascular disease. A systematic review and meta-analysis including dose-response relationship. Scand J Work Environ Health. 2018;44(3):229–38. 29247501 10.5271/sjweh.3700

[9] Ulas T, Buyukhatipoglu H, Kirhan I, et al. The effect of day and night shifts on oxidative stress and anxiety symptoms of the nurses. Eur Rev Med Pharmacol Sci. 2012;16:594–9. 22774399

[10] Gehlert S, Clanton M. Shift work and breast Cancer. Int J Environ Res Public Health. 2020;17(24):9544. 33419321 10.3390/ijerph17249544 PMC7767214

[11] Lin XC, Chen L, Lin RB. Relationship between risk degree of coronary heart disease and serum uric acid and homocysteine [J]. Capital Food Med. 2020;27(16):10–1.

[12] Miake J, Hisatome I, Tomita K, et al. Impact of Hyper- and hypo-uricemia on kidney function. Biomedicines. 2023;11(5):1258. 37238929 10.3390/biomedicines11051258 PMC10215381

[13] Gaubert M, Marlinge M, Alessandrini M, et al. Uric acid levels are associated with endothelial dysfunction and severity of coronary atherosclerosis during a first episode of acute coronary syndrome. Purinergic Signal. 2018;14(2):191–9. 29626320 10.1007/s11302-018-9604-9 PMC5940631

[14] TARNOWSKI M, MALINOWSKI D, SAFRANOW K, et al. MTNR1A and MTNR1B gene polymorphisms in women with gestational diabetes[J]. Gynecol Endocrinol. 2017;33(5):395–8. 28084098 10.1080/09513590.2016.1276556

[15] Uetani M, Suwazono Y, Kobayashi E, et al. A longitudinal study of the influence of shift work on serum uric acid levels in workers at a telecommunications company. Occup Med (Lond). 2006;56(2):83–8. 16267101 10.1093/occmed/kqi178

[16] Sunniva SS, Fossum IN, Bjornvatn B, et al. Personality factors predict sleep-related shift work tolerance in different shifts at 2-year follow-up: a prospective study. BMJ Open. 2013;3(11):e003696. 10.1136/bmjopen-2013-003696 PMC3822308 24189084

[17] Thun E, Bjorvatn B, Osland T, et al. An actigraphic validation study of seven morningness-eveningness inventories. Eur Psychol. 2012;17:222–30.

[18] Torsvall L, Akerstedt T. A diurnal type scale. Construction, consistency and validation in shift work. Scand J Work Environ Health. 1980;6(4):283–90. 7195066 10.5271/sjweh.2608

[19] Zhang W. 1 Yanan Chen,1 and na Chen. Body mass index and trajectories of the cognition among Chinese middle and old-aged adults. BMC Geriatr. 2022;22:613–20. 35870889 10.1186/s12877-022-03301-2 PMC9308927

[20] Makris KC, Heibati B, Narui SZ, et al. Chrono-modulated effects of external stressors on oxidative stress and damage in humans: a scoping review on night shift work. Environ Int. 2023;178:108048. 37463540 10.1016/j.envint.2023.108048

[21] Manzella N, Bracci M, Strafella E, et al. Circadian modulation of 8-oxoguanine DNA damage repair. Sci Rep. 2015;5:13752. 26337123 10.1038/srep13752 PMC4559719

[22] Singh RK, Bansal A. Studies on circadian periodicity of serum and urinary urate in healthy indians and renal stone formers. Prog Clin Biol Res. 1987;227B:305–13. 3628342

[23] Lang F, Greger R, Oberleithner H, et al. Renal handling of urate in healthy man in hyperuricaemia and renal insufficiency: circadian fluctuation, effect of water diuresis and of uricosuric agents. Eur J Clin Investig. 1980;10:285–92. 6775956 10.1111/j.1365-2362.1980.tb00035.x

[24] Singh R, Kumar P, Mishra DN, et al. Effect of gender, Age, Diet and Smoking Status on the circadian rhythm of serum uric acid of healthy indians of different age groups. Indian J Clin Biochem. 2019;34(2):164–71. 31092989 10.1007/s12291-017-0724-8 PMC6486913

[25] Chen Tao C, Changhai R. Analysis of influencing factors of hyperuricemia in physical examination population in Shenyang [ J]. J Clin Military Med. 2020;48(3):267–70.

[26] Tong XL, Zhang LW. Analysis of the characteristics and influencing factors of hyperuricemia in young and middle-aged population [J]. Chin Med J. 2021;56(8):864–8.

[27] Wu Ying L, Rui T, Shaoqiu, et al. Correlation between hyperuricemia and obesity in young college students. Chin J Endocrinol Metabolism. 2020;36(9):773–7.

[28] ZHU Y, PANDYA B J, CHOI HK. Prevalence of gout and hyperuricemia in the US general population: the National Health and Nutrition Examination Survey 2007–2008[J]. Arthritis Rheum. 2011;63(10):3136–41. 21800283 10.1002/art.30520

[29] NAGAHAMA K, ISEKI K, INOUE T, et al. Hyperuricemia and cardiovascular risk factor clustering in a screened cohort in Okinawa, Japan[J]. Hypertens Res. 2004;27(4):227–33. 15127879 10.1291/hypres.27.227

[30] LOHSOONTHORN V, DHANAMUN B, WILLIAMS MA. Prevalence of hyperuricemia and its relationship with metabolic syndrome in Thai adults receiving annual health exams[J]. Arch Med Res. 2006;37(7):883–9. 16971230 10.1016/j.arcmed.2006.03.008

[31] SARI I, AKAR S. Hyperuricemia and its related factors in an urban population, Izmir, Turkey[J]. Rheumatol Int. 2009;29(8):869–74. 19048257 10.1007/s00296-008-0806-2

[32] WANG, XS,ARMSTRONG MEG,CAIRNS, BJ, et al. Shift work and chronic disease: the epidemiological evidence[J]. Occup Med. 2011;61(2):443–4. 10.1093/occmed/kqr001 PMC3045028 21355031

[33] Wang Y, Charchar FJ. Establishment of sex difference in circulating uric acid is associated with higher testosterone and lower sex hormone-binding globulin in adolescent boys. Sci Rep. 2021;11(1):17323. 34462530 10.1038/s41598-021-96959-4 PMC8405811

[34] Rasoulzadeh AA-MY, Mesgari-Abbasi M et al. Nephrotoxic effects caused by co-exposure to noise and toluene in New Zealand white rabbits: a biochemical and histopathological study. Life Sci 2020 Oct 15:259118254. 10.1016/j.lfs.2020.118254 32800833

[35] Ferraro PM, Bargagli M, Trinchieri A, et al. Risk of kidney stones: influence of dietary factors, dietary patterns, and vegetarian-vegan diets. Nutrients. 2020;12(3):779. 32183500 10.3390/nu12030779 PMC7146511

[36] de Pedro Jiménez D, de Diego Cordero R, Romero-Saldaña M, et al. Hyperuricemia in shift workers: a cross-sectional study in a Spanish chemical factory. Rev Esp Salud Publica. 2020;94:e202004028. 32382003

[37] Adane A, Getnet M, Belete M, Yeshaw Y, Dagnew B. Shift-work sleep disorder among health care workers at public hospitals, the case of Sidama national regional state, Ethiopia: a multicenter cross-sectional study. PLoS ONE. 2022;17(7):e0270480. 35802698 10.1371/journal.pone.0270480 PMC9269933

[38] Cha EJ, Bang YR, Jeon HJ, et al. Network structure of insomnia symptoms in shift workers compared to non-shift workers. Chronobiol Int. 2023;40(3):246–52. 36600639 10.1080/07420528.2022.2163654

[39] Rodríguez-González-Moro MT, Rodríguez-González-Moro JM, Rivera-Caravaca JM, et al. Work Shift and Circadian Rhythm as Risk factors for poor Sleep Quality in Public Workers from Murcia (Spain). Int J Environ Res Public Health. 2020;17(16):5881. 32823687 10.3390/ijerph17165881 PMC7459974

[40] Li H, Zhang Y. Effects of physical activity and circadian rhythm differences on the Mental Health of College Students in schools closed by COVID-19. Int J Environ Res Public Health. 2022;20(1):95. 36612417 10.3390/ijerph20010095 PMC9820026

[41] MANDAL A K. MOUNT D B. The molecular physiology of uric acid homeostasis [J]. Annu Rev Physiol, 2015, 77(323 – 45). 10.1146/annurev-physiol-021113-170343 25422986

[42] WANG Y, ZHANG W, QIAN T, et al. Reduced renal function may explain the higher prevalence of hyperuricemia in older people [J]. Sci Rep. 2021;11(1):1302. 33446773 10.1038/s41598-020-80250-z PMC7809022

[43] PEREZ-RUIZ F, CALABOZO M, ERAUSKIN G G, et al. Renal underexcretion of uric acid is present in patients with apparent high urinary uric acid output [J]. Arthritis Rheum. 2002;47(6):610–3. 12522834 10.1002/art.10792

[44] NUGENT C A, TYLER F H. The renal excretion of uric acid in patients with gout and in nongouty subjects [J]. J Clin Invest. 1959;38(11):1890–8. 14427889 10.1172/JCI103966 PMC441775

[45] Yang HJ, Lin HN, Li CN. Prevalence of overweight and obesity in adults in Mentougou District, Beijing City and its relationship to common chronic diseases. Volume 29. Applied Prev Med; Apr.2023. pp. 90–2. 2.

[46] Kanemitsu T, Tsurudome Y, Kusunose N, et al. Periodic variation in bile acids controls circadian changes in uric acid via regulation of xanthine oxidase by the orphan nuclear receptor PPARalpha. J Biol Chem. 2017;292(52):21397–406. 29101234 10.1074/jbc.M117.791285 PMC5766946

[47] Ruskovska T, Beekhof P, Velickova N, et al. Circadian rhythm and time-of-day-effects of (anti)oxidant biomarkers for epidemiological studies[J]. Free Radic Res. 2021;55(7):792–8. 34251957 10.1080/10715762.2021.1942464

[48] Meng X, Huang X, Deng W, et al. Serum uric acid a depression biomarker. PLoS ONE. 2020;15(3):e0229626. 32130258 10.1371/journal.pone.0229626 PMC7055893

[49] Black CN, Bot M, Scheffer PG, et al. Uric acid in major depressive and anxiety disorders. J Affect Disord. 2018;225:684–90. 28917195 10.1016/j.jad.2017.09.003

